# Compromising location privacy through Wi-Fi RSSI tracking

**DOI:** 10.1038/s41598-025-22799-1

**Published:** 2025-11-10

**Authors:** Mariana Cunha, Ricardo Mendes, Yves-Alexandre de Montjoye, João P. Vilela

**Affiliations:** 1 CRACS/INESCTEC and Department of Computer Science, University of Porto, Porto, Portugal; 2 CISUC, Department of Informatics Engineering, University of Coimbra, Coimbra, Portugal; 3 Imperial College London, Exhibition Road, South Kensington, London, UK

**Keywords:** Privacy, Wi-Fi, Received signal strength indicator (RSSI), Fingerprinting risk, Mobile devices, Engineering, Computer science

## Abstract

The widespread availability of wireless networking, such as Wi-Fi, has led to the pervasiveness of always connected mobile devices. These devices are provided with several sensors that allow the collection of large amounts of data, which pose a threat to personal privacy. It is well known that Wi-Fi connectivity information (e.g. BSSID) can be used for inferring user locations. This has caused the imposition of limitations to the access to such data in mobile devices. However, other sources of information about wireless connectivity are available, such as the Received Signal Strength Indicator (RSSI). In this work, we show that RSSI can be used to infer the presence of a user at common locations throughout time. This information can be correlated with other features, such as the hour of the day, to further learn semantic context about such locations with a prediction performance above 90%. Our analysis shows the privacy implications of inferring user locations through Wi-Fi RSSI, but also emphasizes the fingerprinting risk that results from the lack of protection when accessing RSSI measurements.

## Introduction

Smart devices owe their indispensability to the widespread availability and constant improvements in wireless technologies, fostering a society that prioritizes ubiquitous connectivity and mobility. A wide variety of personal mobile devices, such as smartphones, smartwatches, computers, or vehicles, are equipped with a multitude of sensors that allow the collection of massive quantities of sensitive data^1^. These devices also have the capacity to connect to the Internet or to other devices through wireless, which supports the permanently online paradigm. An example of a wireless networking technology is Wireless Fidelity (Wi-Fi) that relies on radio waves to transfer data between devices (e.g. between a wireless Access Point (AP) and a smartphone). The power of the received radio signal can be measured by the Received Signal Strength Indicator (RSSI), where a higher RSSI value corresponds to a stronger signal. The analysis of Wi-Fi signal set the tone for several research works and became popular for indoor localization^2–5^, but also to authenticate devices^6–8^, identify devices or users^9–11^, and infer/monitor human behavior^12,13^.

Despite the utility of exploring RSSI values, the potential threats that arise from the sensitive and/or private information that can be inferred have been neglected^14^. Considering the use of RSSI for indoor localization, it becomes possible to position a user in a specific floor or room within a building^15^. By way of example, this could compromise privacy by disclosing sensitive locations within hospitals where it would be possible to leverage more context about the user and, possibly, about the user’s health.

Regardless of the existing research on RSSI, to the best of our knowledge, we are the first work to investigate the privacy implications of RSSI values and the resulting fingerprinting risk. Towards this goal, we resort to the COP-MODE dataset, where the RSSI measurements were collected from a real-world field study with smartphones. Beyond the fact that these devices are extremely popular and personal, their sensory capacities constitute a rich and pervasive source of private data collection. Moreover, the Wi-Fi RSSI value can be collected without the user perception through permissions that are automatically granted at applications’ install-time. These permissions cannot be denied and are requested in more than 60% of the applications contained in the COP-MODE dataset, which is concerning due to the uniqueness of RSSI. Taking this into account, we define our attack model as a smartphone application that has the mentioned permissions and is capable of collecting the Wi-Fi RSSI measurements.

In order to emphasize the privacy risks of accessing Wi-Fi RSSI, this work demonstrates that RSSI measurements can be used to infer the presence of a person at common locations throughout time. Furthermore, we prove that correlating such data with other features (e.g. hour of the day) can enrich the semantic context of the inferred locations. Our results show that predicting private locations, such as Home/Work, is possible through Wi-Fi RSSI with an F1-Score above 90%. This highlights the privacy implications of granting access to Wi-Fi side information and is a call for an action in revising permissions, but also in raising users’ privacy awareness regarding Wi-Fi privacy threats.

The remainder of this paper is structured as follows. “Background and state of the art” provides an overview of background concepts and presents related works from the state of the art. “Experimental design” describes the experimental design and “Exploratory data analysis” presents the performed exploratory data analysis, including the required permissions to access RSSI values. “Privacy impact of Wi-Fi RSSI” discusses the privacy impact of Wi-Fi RSSI by learning users’ locations and “Conclusion” draws the main conclusions.

## Background and state of the art

The pervasiveness of smart devices and the evolution of Wi-Fi technology has been redefining the paradigm of wireless connectivity. With the growing advances in Wi-Fi performance, a wide range of applications that benefit from sensing the surrounding environment have emerged to provide user-tailored services. Despite being beneficial, their omnipresence in users’ lives can become intrusive and pose serious risks to privacy, such as the possibility of disclosure the users’ identity, beliefs, habits, social relationships, or even health conditions^16^.

Wi-Fi sensing rely on existing Wi-Fi signals to detect events or environmental changes between devices. Based on this, several applications have been proposed and can be grouped into three main categories^17^: activity recognition, object sensing, and localization. Within the activity recognition category, the Wi-Fi signal proved to be able to detect human activities (e.g. running, walking, standing, among others)^12,18–21^, which allowed the development of applications that can monitor users in a non-intrusive manner. As an example, the system proposed in ^21^ monitors vital signs and postures during sleep by exploiting fine-grained Channel State Information (CSI) to capture minute movements caused by breathing and heart beats. Due to the widespread availability of wireless-enabled devices, recent works have also investigated object sensing through wireless^22–24^, such as sensing fruit ripeness ^22^. This example of a sensing system uses Wi-Fi signals and frequency diversity to characterize physiological compounds of the fruit and to sense the physiological changes associated with fruit ripening. The last category is related to localization, which has been a research topic of interest as a result of the high demand for indoor positioning^3,25–28^ and is covered in more detail below. In addition to previous research on Wi-Fi sensing, Wi-Fi signals have also been used as a device-free human identification method, for example, based on the walking gait pattern extracted from the Channel State Information (CSI) ^29^ or by using fluctuations in the Received Signal Strength Indicator (RSSI) ^11^. Nevertheless, these works are limited to a number of users and to a controlled context (e.g. office).

Focusing on wireless indoor positioning, the literature divides existing approaches into geometric and fingerprinting^28,30^. Geometric approaches rely on multitrilateration, trilateration, and triangulation methods to position devices by using measurement parameters, such as Time of Arrival (ToA), Time of Flight (ToF), and Angle of Arrival (AoA), while fingerprinting approaches consider the RSSI (i.e. the received signal strengths from several APs) or the CSI (i.e. a combination of communication link attributes between a transmitter and a receiver) to determine the devices’ positions. Fingerprint-based approaches are typically divided into two phases: offline/training phase and online/positioning phase. In the first phase (offline or training phase), the fingerprints (e.g. signal measurements) are collected and associated with known locations to build a fingerprint database, also known as a radio map. In the second phase (online or positioning phase), the user location is determined by matching the collected fingerprints with the samples in the database. Although fingerprint-based approaches offer a low-cost solution for indoor positioning by leveraging existing Wi-Fi infrastructure, there are still several challenges, including the need to build the initial database (radio map) ^28,31^, which is highly susceptible to environmental changes and requires frequent recalibration.

In the context of wireless indoor positioning, fingerprinting corresponds to a positioning approach, where the concept of fingerprint consists of signal measurements collected by a device that will be used to match a location based on given samples. However, the fingerprint concept is generically referred to as digital fingerprinting, also known as device fingerprinting, which corresponds to a technique used to uniquely identify and track devices/users. Digital fingerprinting relies on a feature or combination of features that uniquely identify an individual. Throughout this paper, the fingerprint concept will be used based on this latter definition, that is, a feature or combination of features (e.g. RSSI measurements) that can be used to uniquely identify and/or track devices, which differentiates our work from existing ones.

Opposed to CSI, RSSI is widely available from various wireless networks and does not require a special device or infrastructure to be accessed ^11^. In particular, collecting RSSI measurements is possible through a smartphone application without constraints. Regardless of the developments in permission managers, the current smartphone permission model still has limitations and fails to account for data correlation and contextual dependency ^32,33^. For example, Wi-Fi data might disclose location-related information ^34,35^, which has privacy implications that were underestimated in previous Android versions ^36^. In recent years, numerous data breaches have been reported^1,37^ and, as a result, the privacy of countless users has been compromised. Due to the uniqueness of the data, re-identifying the identity of the users is often possible even in anonymized datasets^38^. As an illustration, human mobility traces are highly unique, and four spatio-temporal points from Wi-Fi, GSM, and GPS traces revealed to be sufficient to uniquely identify above 90% of the users^39^.

From the perspective of Wi-Fi APs (i.e. non user-centric), several privacy concerns have been raised, since these devices are often used to track and/or to fingerprint users by monitoring probe requests and collecting inherent information sent by users’ devices ^40–42^. Both smartphones and laptops periodically send Wi-Fi probe requests either broadcast (i.e. not specifically directed to a Wi-Fi network) or directed (i.e. specifying the SSID) containing the MAC address that uniquely identifies the sending device. From the collected data, it is possible not only to track the users, but also to use the available data to infer information about the users that are typically in the range of that AP ^40,42–44^. MAC address randomization emerged in response to these privacy violations by replacing the hardware-based MAC address (i.e. a device static identifier) with a temporary/randomized one, thus preventing third parties from tracking devices. Our paper examines a different perspective (user’s perspective) of accessing Wi-Fi data through an application installed on the user smartphone, emphasizing the privacy implications of a user-centric attack model that has access to the available unconstrained RSSI information to infer user’s common location.

Building on this knowledge, our work differs from previous studies by quantifying for the first time the privacy implications of accessing Wi-Fi RSSI measurements and available information (e.g. hour of the day) from a smartphone application without restrictions. Despite the prior research on Wi-Fi signal and, specifically, on the development of methods for indoor-positioning, where a relative positioning to known locations is required to build a radio map, our approach explores the re-identification of users’ locations without any offline/radio map. In contrast to approaches that rely on background information about reference points, such as locations of Wi-Fi APs, we consider a user-centric attack model (i.e. an application within the user’s smartphone) that collects the RSSI measurement. In our paper, we further analyze the privacy impact of inferring a user common location through Wi-Fi RSSI, as well as the issues that advent from enriching such data and exploring existing correlations. Moreover, we take advantage of the fact that a smartphone application with install-time permissions (i.e. automatically granted by the system) is capable of collecting Wi-Fi RSSI measurements without user perception to emphasize the need to protect such information.

## Experimental design

Wi-Fi RSSI measures the received signal strength from a wireless device. Smartphones, an example of a popular personal mobile device, allow the pervasive collection of RSSI measurements without the user perception, which raises privacy concerns. This section starts by defining the problem and describing the dataset that was used as a proof of concept to support our findings. Throughout this section, smartphone application might be referred to as app.

### Problem definition and attack model

The sensory capacities of smartphones and the pervasiveness of services and mobile applications constitute a rich source of personal data. The privacy implications of collecting such data fostered a set of protection mechanisms to address the inherent issues, including the development of new permissions. For example, recent Android versions require location-related permissions to access the BSSID (MAC address) and the SSID (network name) of nearby Wi-Fi Access Points (APs), and Android 13 introduced the NEARBY_WIFI_DEVICES permission with a location attribute^45^. With these permissions, Android tightened the access to Wi-Fi information by applying restrictions similar to those applied to access to location data, requesting a user’s answer through a permission prompt. To access location, there are two main permissions: ACCESS_COARSE_LOCATION, that provides an approximated estimation within about 3 square kilometers, and ACCESS_FINE_LOCATION, that provides a precise estimation usually within about 50 m and sometimes as accurate as within 3 m or better^46^. Despite these location-related constraints, an application capable of scanning nearby Wi-Fi devices can still extract sensitive information about the user’s location by accessing the BSSID (MAC address) and the SSID (network name) of nearby Wi-Fi APs ^34^.

In addition, although these enhancements have been released to mitigate the possible inferences that advent from data correlations, specifically the extraction of user’s location through Wi-Fi data^34,36^, accessing the Wi-Fi RSSI is still possible through a smartphone application with non-dangerous permissions, which are automatically allowed by the system when the application is installed and cannot be denied. This raises privacy risks that will be tackled in this work. Towards this goal, we define our attack model as a smartphone application that is capable of collecting the RSSI of Wi-Fi at a certain timestamp. This information might be obtained in runtime from the current connection without the user perception. Any entity with access to the collected data is assumed as an adversary that might attempt to make private inferences about users. Taking this user-centric adversary model into account, the objective is to demonstrate the privacy impact of accessing Wi-Fi RSSI by disclosing common locations of users. During the performed analysis, we will also assume a stronger adversary that is able to learn the semantic of the users’ locations by enriching and correlating the Wi-Fi RSSI data with background information, such as the hour of the day.

### Dataset characterization

**Fig. 1:**
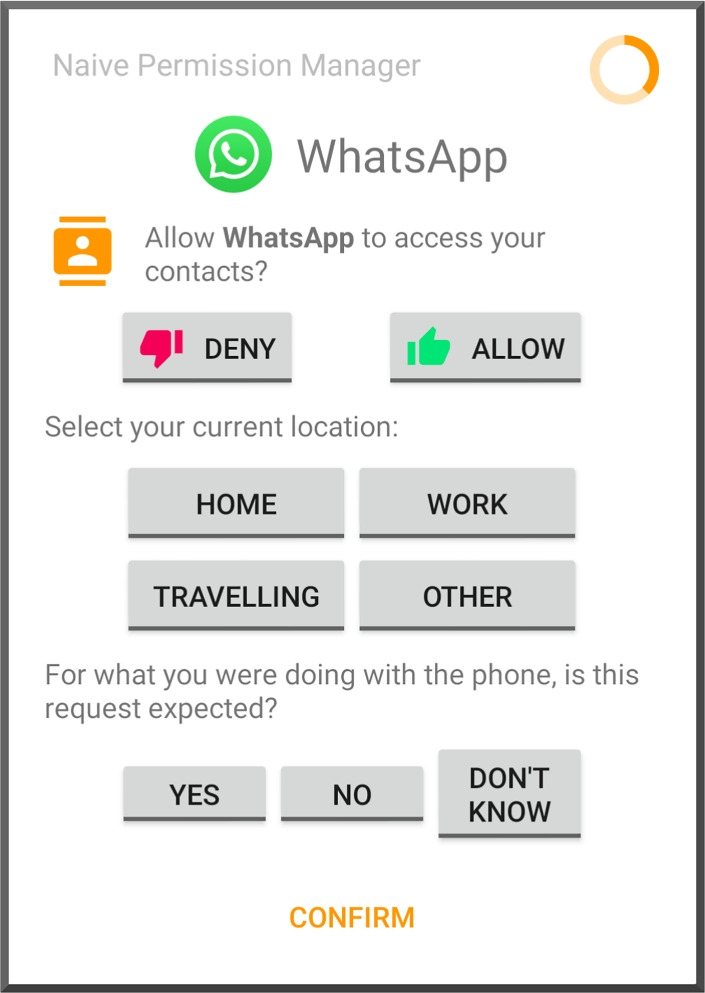
An example of a permission prompt issued as a result of the app *WhatsApp* checking for the contacts permission (from ^32^).

As a proof of concept, this paper resorts to the COP-MODE dataset from a field study conducted with 93 participants ^32^. This field study was approved by the Ethics Committee of the Department of Computer Science and Technology of the University of Cambridge and by the Ethics Commission of the Faculty of Sciences of the University of Porto. The participants carried smartphones with their personal applications pre-installed and an application responsible for data collection for a period of at least one week. This app prompts users at every permission check and collects their input, as well as other contextual features at the time of the prompt (see Fig. 1). The smartphone used by the participants was the Pocophone F1 with a custom ROM (Read-Only Memory) based on the Android Open Source Project (AOSP), named PixelExperience (version Android 9.0).

The resulting dataset, which is further analyzed in ^32^, is composed of 93 volunteers from Portugal who were recruited by word of mouth, university mailing lists, and oral presentations. This resulted in the participation of 60 (64.5%) students, 11 (11.8%) researchers, and 19 (20.4%) with diverse backgrounds. Regarding demographics, 66 (71%) participants were 18–24 years old, 68 (73.1%) were males, and 53 (57%) with an Information Technology (IT) background (studying or professionals). Therefore, the dataset is skewed towards young adults and slightly more than half with an IT background. However, the findings of this paper hold more generally, since the percentage of the global population that uses smartphones is approximately 90%, with Wi-Fi dominating the usage of Android mobile data ^47^. Due to the limitations in existing datasets, an anonymized version of the COP-MODE dataset is available to interested researchers ^48^, as detailed in the Data Availability statement.

While the dataset contains more data ^32^, we focus on specific contextual features that are of relevance to this work and collected at the time of each prompt (see Fig. 1). In particular, we selected the following features:

- Datetime: timestamp of the request permission prompt.
- Network status: *disconnected*, *metered* or *unmetered*.
- Wi-Fi: timestamp, BSSID, SSID, and RSSI for each scanned device.
- Semantic location: semantic location was collected from the user input with limited possibilities, including *home* and *work*.

The network status represents the current connection of the smartphone, whose possibilities are: *disconnected*, if there is no Internet connection, *metered*, if the user is connected to a mobile data network, and *unmetered*, if the user is connected to a Wi-Fi network. With respect to Wi-Fi data, the scanned Wi-Fi devices correspond to the devices in the neighborhood that were obtained from a scan attempted every 5 min. These considerations will be taken into account during the exploratory data analysis.

## Exploratory data analysis

The COP-MODE dataset is composed by 2,180,302 permission requests from 93 participants. 52,590 (2.41% of the total requests) have Wi-Fi information from a total of 82 participants. From the 52,590 requests, 43,986 (83.64% of the considered requests) are connected to an unmetered network (i.e. Wi-Fi network), 7301 (13.88% of the considered requests) are connected to a metered network (i.e. mobile data network), and only 1303 (2.48% of the considered requests) are disconnected. At each permission request, the COP-MODE dataset collected the network status of the smartphone, as well as the list of scanned Wi-Fi devices, their RSSI, and the semantic location selected by the user. We preprocess the scanned Wi-Fi devices list into rows (one per AP) containing the following features: user, timestamp of the request, network status, selected semantic location, timestamp of Wi-Fi scanning, Wi-Fi BSSID, Wi-FI SSID, and Wi-Fi RSSI. After removing the duplicated rows, we obtained the data that constitutes the target of analysis in this paper and, hereafter, COP-MODE dataset refers to this data.

### Permissions requested to obtain Wi-Fi RSSI

The ease of access to the RSSI value through a smartphone application was one of the motivations of this work. Taking this into account, we start by studying the context of installed applications with respect to the permissions required to access Wi-Fi RSSI. This information results from a preliminary step of the field study campaigns, where the personal apps and respective permissions were collected from the participants’ smartphones. The applications are divided into system and non-system apps, with a total of 3926 distinct apps (out of 30,768 installed apps) and 1737 non-system distinct apps (out of 5315 installed apps).

In order to control applications’ access to sensitive resources/data, smartphones rely on a permission system where users can grant or deny permissions and, consequently, grant or deny the resources/data that can be accessed by each application. In the Android mobile operating system, these permissions are divided into install-time, runtime, and special permissions^49^. The special permissions are defined only by the platform or Original Equipment Manufacturer (OEM). The install-time permissions are automatically granted by the system when the application is installed, while the runtime permissions, also known as dangerous permissions, need to request a permission prompt the first time the application requires the permission. After being accepted once, the permission is granted until explicitly disabled in the device settings.

As previously discussed, the privacy implications of collecting certain data types have led to the development of new permissions and/or new restrictions to the data collection. Nevertheless, Wi-Fi RSSI data is still possible to obtain through the following non-dangerous permissions: ACCESS_WIFI_STATE or ACCESS_NETWORK_STATE. These permissions allow applications to access information about the Wi-Fi networks (including RSSI) and all networks, respectively. From the performed analysis, 1499 ($\approx$ 38%) apps request the permission ACCESS_WIFI_STATE corresponding to a total collection of 10,402 (3401 from non-system apps) and 2419 ($\approx$ 62%) apps request the permission ACCESS_NETWORK_STATE corresponding to a total collection of 15,609 (5069 from non-system apps). In summary, at least one of these permissions is requested in about 63% of the applications, which, as aforementioned, are categorized as install-time and automatically granted without any interaction from the user.

**Fig. 2:**
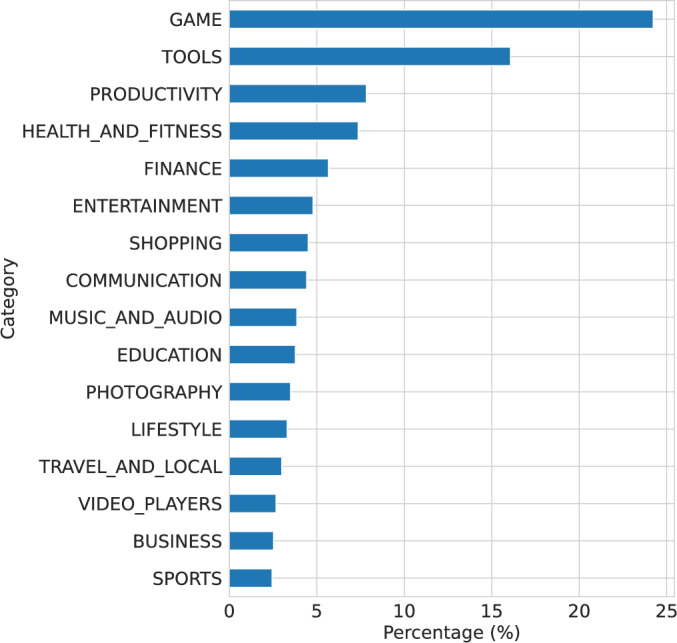
Percentage of distinct apps category in which the permissions ACCESS_WIFI_STATE and/or ACCESS_NETWORK_STATE were requested. Categories with a percentage of apps inferior to 1% were removed from the plot to simplify visualization.

To better understand the applications that request these permissions, we relied on the COP-MODE dataset and categorized the applications according to the Google Play Store. Figure 2 presents the percentage of distinct apps from each category in which the permissions ACCESS_WIFI_STATE and/or ACCESS_NETWORK_STATE were requested. For illustration, almost 25% of the apps requesting these permissions are in the GAME category. Although some categories are expected to request the ACCESS_WIFI_STATE and/or ACCESS_NETWORK_STATE permissions due to the app objectives, this analysis shows the diversity of applications that request and have automatically granted access to network-related permissions independently of the app category and the users’ privacy preferences.

### Wi-Fi RSSI analysis

**Fig. 3:**
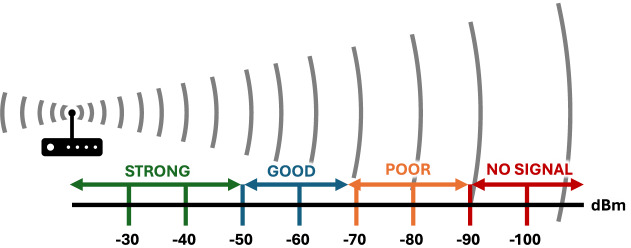
Representation of RSSI values based on the distance to the Wi-Fi AP and to the quality of the signal.

As aforementioned, the Wi-Fi signal strength can be represented as an RSSI value that is commonly expressed in decibels relative to a milliwatt (dBm). This value measures how well a device can hear a signal from a correspondent wireless Access Point (AP), which allows to determine if the signal is sufficient to establish a good wireless connection. The range of RSSI is not standardized and depends on the adapter vendor, typically ranging from 0 to − 120 dBm, where a stronger signal corresponds to values closer to 0. The RSSI value can be correlated to the quality of the signal and to the AP proximity, which is visually represented in Fig. 3. According to^50^, an RSSI value lower than − 90 dBm corresponds to an extremely weak connection, a weak connection ranges between − 66 dBm and − 90 dBm, a good connection is established between − 51 dBm and − 65 dBm, a strong connection ranges between − 50 dBm and − 31 dBm, and an extremely strong connection has a value higher than − 30 dBm, commonly − 30 dBm to − 20 dBm.

**Fig. 4:**
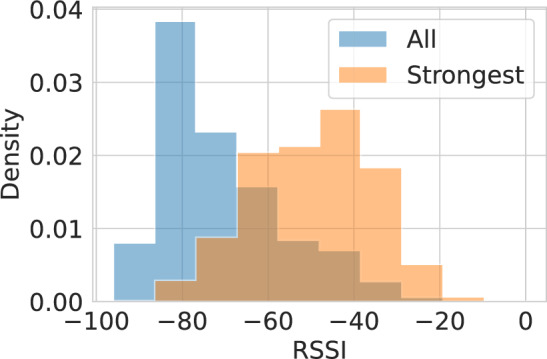
Comparison between the Wi-Fi RSSI density of all scanned Wi-Fi APs and the Wi-Fi RSSI density of the strongest APs (i.e. the AP the user would be connected to). The density means that the histogram is normalized so that the total area under the bars is 1.

In real world, smartphones scan several Wi-Fi APs with different values of signal strength. From the available data, we were able to analyze the diversity of RSSI values that are scanned by smartphones in a real use case. Figure 4 shows the density distribution of the RSSI values according to two scenarios: (1) *All*, that is, the RSSI values of all scanned Wi-Fi APs and (2) *Strongest*, that is, the Wi-Fi RSSI density of the strongest APs, which represents the AP the user would be connected to^51^. The density means that the histogram is normalized so that the total area under the bars is 1. From the results, it is possible to understand the differences between the distributions and, specifically, the lower values when considering all of the scanned APs. These results are in accordance with the previously presented relation between the signal strength and the RSSI values, demonstrating that even if the signal is weak to establish a wireless connection, a Wi-Fi AP might be scanned by a smartphone. From a privacy perspective, the more available scanned APs, the more enriched inferences would be possible to obtain about the users. These scenarios will be considered throughout the performed analysis.

## Privacy impact of Wi-Fi RSSI

Motivated by the ease of access to Wi-Fi RSSI measurements, we now tackle the privacy implications of collecting such data. Recalling our attack model, this section demonstrates that an attacker able to collect Wi-Fi RSSI values can compromise users’ privacy by inferring common locations, but also learning the locations’ semantic context. As aforementioned, the COP-MODE dataset was collected with a smartphone application from a field study with users in real world. Apart from data obtained through smartphone sensors, this dataset contains the semantic location from the user input. Taking advantage from having this data as ground-truth, we demonstrate next the inferences that an attacker could make through the Wi-Fi RSSI.

### Inferring user common locations

Humans tend to be repetitive throughout the time, as a consequence of daily routines. Such repetitiveness has posed a limit to users’ privacy^52^ by allowing not only to track users over time, but also to predict a future behavior. Building on this, we started by evaluating the privacy impact of using Wi-Fi RSSI to infer the presence of a user at common locations.

Since users tend to frequent the same places during their days, the Wi-Fi APs they connect to can be associated to specific locations. Despite the variability of the RSSI, similar sets of RSSI values are likely obtained for the same locations, thus enabling identification of common locations from RSSI measurements as we shall demonstrate. Similarly, a person who is at work at 9am on a Monday is expected to be at work at 9am on a Tuesday. Therefore, we evaluate the privacy impact of inferring the presence of a user at a common location by considering the measured RSSI, the hour of the day, and the combination of both features.

**Fig. 5:**
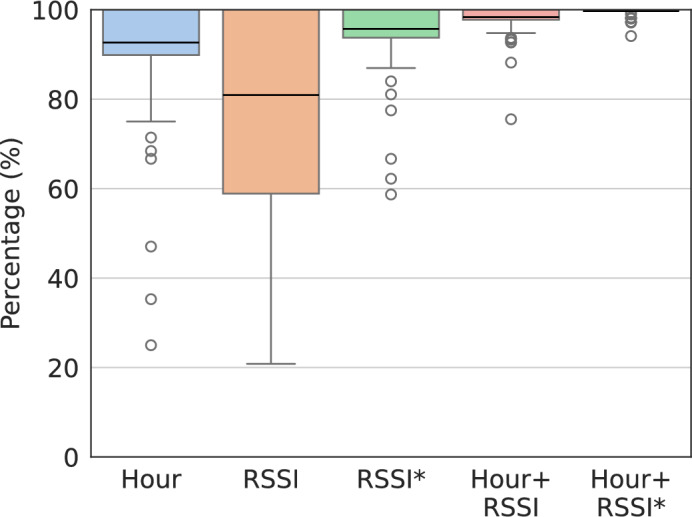
Percentage of common locations that were correctly inferred from: *Hour*, that is, the hour of the day, *RSSI*, that is, the Wi-Fi RSSI collected from all the scanned Wi-Fi APs, RSSI*, that is, the Wi-Fi RSSI collected from the strongest Wi-Fi AP, and the combination of both features. The black line represents the mean value.

Figure 5 presents the percentage of common locations that were correctly inferred from the hour of the day, the Wi-Fi RSSI, and the combination of both features. A common location is correctly inferred when the feature or combination of features were reported in only one location. In the performed analysis, we resorted to the previously presented scenarios where the attacker would access (1) RSSI measurements from all the scanned Wi-Fi APs, or (2) RSSI of the strongest scanned Wi-Fi AP, which is represented as RSSI* in the chart. From these results, we observe the possibility of inferring that a user is frequenting the same location through the hour of the day and the RSSI value. In particular, the hour of the day highlights the routinary behavior of people and, hence, poses a threat to location privacy. In regard to the RSSI value, although the values collected from all scanned Wi-Fi APs introduce variability in this feature, RSSI is still able to disclose a location, in average, about 80% of the cases. On the other hand, the RSSI collected from the strongest Wi-Fi AP (RSSI* in the chart) revealed to be sufficient to disclose the user’s location over 90% of the times. The overall best result is achieved when correlating the hour of the day with the RSSI* (i.e. the RSSI collected from the strongest Wi-Fi AP). These conclusions reiterate the privacy concerns of accessing Wi-Fi RSSI and the possibility of inferring when the user is at a common location.

### Learning semantic locations

In addition to infer a user common location, we now demonstrate that an attacker with background information (e.g. Wi-Fi BSSID, Wi-Fi SSID or nearby Points-of-Interest) would be able to learn the semantic of such location through the Wi-Fi RSSI. Recalling Machine Learning (ML) approaches used in the context of indoor localization^53,54^, we now follow this line and model our goal as a classification problem to illustrate a possible attack. Taking advantage of the fact that the COP-MODE dataset contains data labeled with semantic location, we relied on an Automated Machine Learning (AutoML) framework to do the selection and hyperparameter tuning of the classification model that better fits our problem. From a thorough comparison of existing AutoML frameworks ^55–57^, we selected Auto-Sklearn ^58^, an open-source AutoML framework, with high popularity due to its compatibility with scikit-learn, that achieves the best performance for classification problems ^55^. Auto-Sklearn compares several classifiers (e.g. Random Forest, Ada Boost, k-Nearest Neighbors, Linear Discriminant Analysis, among others) and predicts with the one that achieves the best overall results. In this case, we aim at predicting the semantic location (Home/Work) through Wi-Fi RSSI measurements. Beyond using these values, we follow the conclusions of the above analysis and consider the timestamp of data collection, specifically, the hour of the day as a feature.

The Auto-Sklearn classifier was trained with 75% of the data with the following features: the Wi-Fi RSSI measurements, the hour of the day, and the combination of both features. Since participants spent the majority of the time at home, the classes (Home/Work) in the dataset are imbalanced. Although the difference between the time spent at Home/Work is expected and increasingly common due to remote work, we tackle the performed analysis with both imbalanced and balanced data. As a preparation step, we started by testing some methods to balance the classes (Home/Work), achieving the best result through the oversampling method. This method allows to adjust the class distribution of data by randomly duplicating samples of the minority class.

**Fig. 6:**
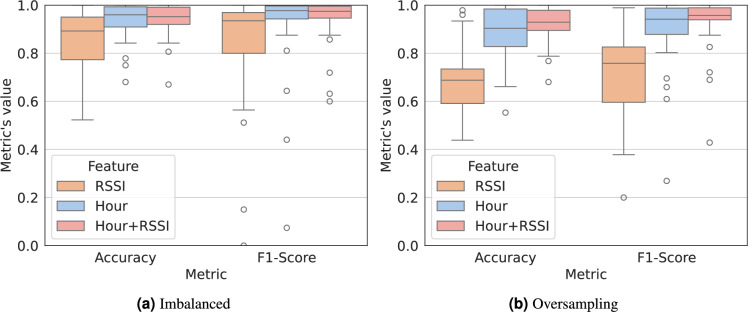
Boxplot with model’s performance based on the Accuracy and F1-Score metrics, where 0 corresponds to a poor performance. The features considered are: *RSSI* (i.e. Wi-Fi RSSI collected from all the scanned Wi-Fi APs), *Hour* (i.e. hour of the day), and the combination of both features. The boxplot line represents the median value.

To evaluate the model’s performance, we consider the Accuracy and F1-Score metrics. The Accuracy metric assesses the model behavior by quantifying the number of correctly predictions made. The F1-Score corresponds to the harmonic mean of the precision and recall, while taking into account both the true/false positives/negatives. This evaluation is presented in Fig. 6 both to the imbalanced (Fig. 6a) and to the balanced with oversampling (Fig. 6b) predictions. These metrics range from 0 to 1, where 0 corresponds to a poor performance. From the results, we can conclude about the quality of the hour of the day as a feature, which has an average Accuracy and F1-Score of about 90%. Correlating the Wi-Fi RSSI information with the hour of the day allows us to infer the semantic location and, thus, assign a context to the identified location. In fact, the timestamp information is available to a smartphone application and, therefore, can be combined with RSSI to enhance the learning outcome without additional permissions.

**Fig. 7:**
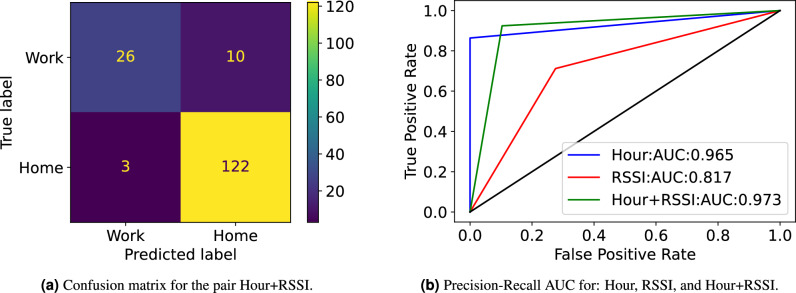
Detailed analysis of the model’s performance for a certain user, for each considered feature: (Hour) hour of the day, (RSSI) Wi-Fi RSSI measurement, and (Hour+RSSI) the combination of both features.

For illustration, Fig. 7 details the analysis for a certain user by presenting: the confusion matrix for the Hour+RSSI features (Fig. 7a) and the Precision-Recall AUC (Area Under the Curve) for each feature (Fig. 7b). The confusion matrix summarizes the performance of the ML model applied to test data and measures the performance of the classification model through the number of True Positives (TP), True Negatives (TN), False Positives (FP), and False Negatives (FN). The Precision-Recall AUC represents how well the model is capable of distinguishing between classes (i.e. Home/Work in our case). These results support the possibility of inferring the semantic of locations with a Precision-Recall AUC of about 97% when correlating both features: hour of the day and RSSI. This is specially concerning due to the uniqueness of human mobility and resulting re-identification risk.

## Conclusion

In this digital age, the everyday-carried smart devices are almost always equipped with wireless technologies, such as Wi-Fi. On par with other Wi-Fi-enabled devices, smartphones can continuously send and/or receive signals that can pose threats to privacy. Although the advances in the permission managers and the constraints employed when dealing with Wi-Fi data, the RSSI measurements can be obtained through a smartphone application without user perception. Accessing the Wi-Fi RSSI value is still possible through the ACCESS_WIFI_STATE or ACCESS_NETWORK_STATE permission, that is, install-time permissions automatically granted by the system that cannot be denied. In this paper, we evaluate the privacy impact of inferring users’ location through Wi-Fi RSSI measurements collected with a smartphone application. By correlating RSSI information with other features such as the hour of the day, we demonstrate the possibility of identifying user common locations as well as learning their semantic information with a performance above 90%. In addition to predict user common locations and respective semantic context, these inferences could be mapped with location information about access points to identify the user location coordinates, a line we plan to explore as future work.

## Data Availability

An anonymized version of the dataset is available to interested researchers^48^. Please contact us for access or for potential collaborations. All shareable data is stripped of identifiable information.
